# Immobilization of Lipases on Chitosan Hydrogels Improves Their Stability in the Presence of the Products of Triglyceride Oxidation

**DOI:** 10.3390/gels9100776

**Published:** 2023-09-24

**Authors:** Domenico Pirozzi, Alessandro Latte, Filomena Sannino

**Affiliations:** 1 Laboratory of Biochemical Engineering, Department of Chemical Engineering, Materials and Industrial Production (DICMaPI), University of Naples Federico II, Piazzale Tecchio 80, 80125 Naples, Italy; domenico.pirozzi@unina.it (D.P.); alessandro.latte@gmail.com (A.L.); 2 Department of Agricultural Sciences, University of Naples Federico II, Via Università 100, Portici, 80055 Naples, Italy

**Keywords:** biopolymer, cross-linking, chitosan hydrogel, adsorption, entrapment, lipase

## Abstract

A significant bottleneck for the industrial application of lipases stems from their poor stability in the presence of commercial triglycerides. This is mainly due to the inactivating effect of the products of triglyceride oxidation (PTO), which are usually produced when oils and fats, being imported from far countries, are stored for long periods. In this study, the immobilization of a lipase from *Candida rugosa* on chitosan hydrogels has been carried out following two alternative approaches based on the enzyme adsorption and entrapment to increase the lipase stability under the operating conditions that are typical of oleochemical transformations. The effect of model compounds representing different classes of PTO on a lipase has been studied to optimize the enzyme immobilization method. Particular attention has been devoted to the characterization of the inactivating effect of PTO in nonaqueous media, which are adopted for most industrial applications of lipases.

## 1. Introduction

Microbial lipases have been increasingly used in the last decades [1] as they make possible the development of green, sustainable processes adopting mild reaction conditions and simpler synthetic routes, with minimized amounts of by-products and wastes generated, and improved regio-, chemo- and stereo-selectivities. In addition, immobilized enzymes show increased thermal and operational stability and offer simpler methods for their recovery and reuse [1].

In describing the industrial applications of lipases, it is necessary to make a distinction between hydrolytic reactions (usually carried out in aqueous media) and synthetic reactions (generally performed in nonaqueous media).

The hydrolytic reactions of lipases are exploited in environmental applications, where lipid-based wastes are broken down and degraded in the pulp and paper industry [2]. In addition, these reactions are used in the leather industry to reduce the fraction of resinous substances and modify the surface properties of fibers [3] to aid in the dehairing and degreasing of animal hides before the tanning process. Furthermore, lipase-catalyzed hydrolytic reactions are exploited in the food industry [4] to modify the taste and aroma of foods by breaking down lipid molecules into different fatty acids.

- The synthetic reactions of lipases are related to the modification of triglycerides and their derivatives due to their potential to catalyze different reactions, the mild conditions required (low temperature and no acid catalysts) and the reduced requirements for the downstream purification of the products [5]. In particular, lipases can be used in the oleochemical industry to obtain specialty or dietary oils, in the production of biodiesel by esterification of triglycerides with methanol, in the pharmaceutical industry to obtain lipid-based drug formulations or lipid nanoparticles containing drugs, or in the manufacture of emulsifying agents [6], cosmetics, soaps [7], and shampoos to modify the lipid content and improve the texture and performance of high-quality products.

Unfortunately, a significant bottleneck for the industrial application of lipases stems from their poor stability in the presence of commercial triglycerides [8], which is mainly due to the inactivating effect of the products of triglyceride oxidation (PTO). Many studies have shown that the oxidation of commercial triglycerides leads to the formation of primary oxidation products, mainly fatty acid hydroperoxide isomers, that may eventually generate a complex mixture of secondary oxidation products [9]. Most of these products are reactive oxygen species (ROS), such as superoxide radicals (O_2_^−^), hydrogen peroxide (H_2_O_2_), and hydroxyl radicals (OH^−^). They are highly reactive and may lead to enzyme inactivation through oxidative stress [10]. As a matter of facts, ROS can react with amino acid residues in enzymes, leading to oxidative modifications, such as the oxidation of sulfur-containing amino acids (cysteine and methionine) and the formation of protein carbonyls. These modifications can disrupt the enzyme’s active site, structure, and function, causing changes in the three-dimensional structure of the enzyme and eventually, its denaturation. Denatured enzymes lose their catalytic activity and may even aggregate, rendering them nonfunctional. The generation of these products occurs in many instances, as oils and fats are often imported from very far countries and are consequently stored for long periods [10].

The immobilization of lipases may offer a potential approach to reduce these inactivating effects [11]. Chitosan, a biocompatible and biodegradable polysaccharide, made from D-glucosamine and *N*-acetyl-D-glucosamine, is increasingly used as a support for enzyme immobilization. It is worth noting that chitosan is obtained from alkaline or enzymatic *N*-deacetylation of chitin, a residual product of shrimp and seafood processing [12]. Consequently, the use of chitosan is consistent with the principles of the circular economy, in that the conversion of chitin into chitosan prevents the disposal of chitin as a waste. As a matter of facts, the annual production of chitin is about 1 × 10^12^ tonnes, but its industrial application is limited by the low solubility in an aqueous solution [13] which makes difficult its interaction with other compounds.

Chitosan has been recently used for applications in the fields of food, medicine, cosmetics, and wastewater treatment due to its properties [14] such as biocompatibility, low cost, nontoxicity, mechanical stability, antimicrobial activity [15], and biodegradability [16]. Chitosan has raised a growing interest as a sorbent material, mainly because it offers high adsorption efficiency due to its hydroxyl groups (–OH) and primary amine groups (–NH_2_) that may act as adsorption sites [15]. These functional groups also improve the interaction of chitosan with different compounds, allowing the synthesis of different composite materials showing interesting properties. The applicability of chitosan has been extended by adding different additives [17] to obtain composites or hybrids. In this way, it has been possible to improve its surface area, to extend the range of pollutants potentially adsorbed, and to allow an easier separation from the aqueous phase (e.g., producing magnetic chitosan by interaction with Fe_3_O_4_).

In particular, chitosan hydrogels possess high adsorption capacity, and improved mechanical, thermal, and porosity properties [18]. Due to their unique properties and wide-ranging applications, these hydrogels have gained significant attention in various fields. The production of chitosan hydrogels involves the cross-linking of chitosan molecules to form a three-dimensional network structure that can absorb and retain large amounts of water. This network of interconnected polymers gives the hydrogel its characteristic gel-like consistency and high water content. Cross-linking can be achieved through various methods, including chemical, physical, or enzymatic cross-linking, depending on the intended application. The relevant properties of chitosan hydrogels are biocompatibility, and consequently the suitability for biomedical and pharmaceutical applications, and biodegradability, which is beneficial for various medical and environmental applications. As a consequence, the chitosan hydrogels are already used in a range of commercial applications, such as drug delivery, being able to encapsulate and release drugs in a controlled and sustained manner protecting them from degradation; wound dressings, as they can promote wound healing and create a moist environment that aids in tissue regeneration and reduces the risk of infection; tissue engineering, as they can create scaffolds that support cell growth and tissue regeneration mimicking of the extracellular matrix, and provide a suitable environment for cell attachment and proliferation; cosmetic and personal care products, such as moisturizers and facial masks, due to their ability to retain water and provide a hydrating effect on the skin; agricultural applications, as they improve water retention and nutrient delivery in soil, protecting plants from pathogens and pests; and the environmental remediation, due to their ability to adsorb heavy metals and organic pollutants from water sources.

Chitosan hydrogels have also been used as matrices to immobilize different enzymes [19], in particular lipases [20]. As a matter of fact, chitosan hydrogels may improve the loading of the enzyme, as well as their catalytic activity and stability, due to their large surface area, their elasticity, and their tunable functional properties [21].

This study is aimed at developing a method to increase the lipase stability in the presence of PTO, based on the immobilization of lipases on chitosan hydrogels. The potential offered by immobilized lipases will be used to improve the stability of the enzyme under the operating conditions that are typical of oleochemical transformations. Particular attention has been devoted to reactions in nonaqueous media [8], which are adopted for most industrial applications of lipases. As a matter of fact, using lipases in nonaqueous media (i.e., organic solvents or other non-water-based environments) offers several advantages and opens up new possibilities for various industrial applications.

The use of lipases in nonaqueous media is advantageous for different reasons. First of all, it should be considered that several lipases are prone to inactivation in under aqueous conditions, whereas in nonaqueous media they exhibit increased stability and longevity, allowing for longer reaction times and higher enzyme reusability [22]. In addition, nonaqueous media can alter the enzyme’s selectivity and specificity, enabling lipases to catalyze specific reactions that may not occur or would be less efficient in aqueous environments, to obtain specific products with high purity and yield [23]. Lipases may have a broader substrate scope in nonaqueous media, as some substrates might not be water-soluble but can be accommodated by lipases in organic solvents. This expands the range of potential reactions and applications [22]. Nonaqueous media often allow higher reactant concentrations, thus enabling process intensification and higher reaction rates, which is of critical importance in large-scale industrial applications [24]. Finally, it is important to consider that some lipases may have difficulty accessing and interacting with hydrophobic substrates in aqueous environments, and nonaqueous media can be adopted to overcome this problem [24].

In this view, the effect of the autoxidation products of triglycerides on the stability of a lipase from *Candida rugosa* was studied to provide useful indications for their removal or chemical neutralization. The immobilization of lipases in chitosan hydrogels has been carried out following two alternative approaches based on enzyme adsorption and entrapment, respectively.

## 2. Results and Discussion

### 2.1. Adsorption of Lipase on Chitosan

**Figure 1:**
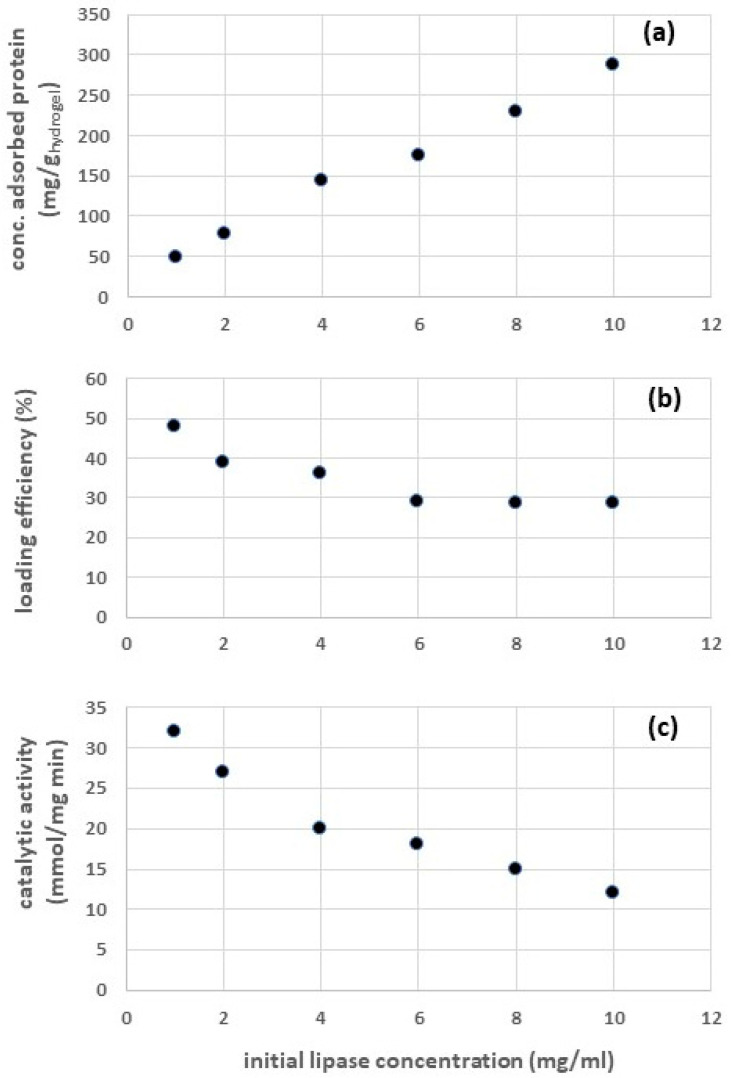
Results of lipase adsorption on chitosan hydrogel particles at different values of the initial lipase concentration: (**a**) concentration of the adsorbed enzyme, (**b**) loading efficiency, and (**c**) catalytic activity.

Chitosan hydrogel particles with adsorbed lipases (CH-ADS) were studied in the first series of experimental tests. Figure 1a,b show the concentration of adsorbed protein per unit weight of hydrogel and the loading efficiency obtained after a gentle stirring for 24 h at 4 °C as a function of the initial protein concentration. The experimental data show that higher values of concentration of the adsorbed protein (from 48 to 287 mg protein per g hydrogel) are observed as the initial lipase concentration is increased (from 1 to 10 mg/mL). Meanwhile, the loading efficiency decreased from 48% to 29%. When the initial lipase concentration was raised above 6 mg/mL, the loading efficiency did not change significantly.

There are a few reasons why the loading efficiency is lower when the enzyme concentration is increased:

- Adsorbent supports have a limited number of binding sites available for enzyme molecules to attach [25]. As the enzyme concentration increases, the number of available binding sites on the support becomes insufficient to accommodate all the enzymes, leading to reduced loading efficiency. At a certain point, the adsorption sites become saturated, and further increases in enzyme concentration do not result in a proportional increase in enzyme loading.
- The reaction system may contain multiple compounds binding to the same adsorption site simultaneously [26]. This competitive adsorption can reduce the efficiency of individual enzyme binding, resulting in lower overall enzyme loading efficiency.
- High enzyme concentrations can lead to increased aggregation or self-interaction of enzymes in the solution [25]. Aggregated enzymes may have limited access to the adsorbent surface or may be less likely to effectively interact with the adsorption sites, reducing their loading efficiency.
- At higher enzyme concentrations, the local concentration of enzymes near the adsorption sites may become very high, leading to hindered diffusion of enzymes to the adsorbent surface [25]. Hindered diffusion can slow down the rate of enzyme attachment to the adsorbent, reducing the overall loading efficiency.

Consequently, to optimize enzyme loading on an adsorbent support, it is essential to carefully balance enzyme concentration, adsorbent properties, and the process conditions, to achieve the highest possible loading efficiency while avoiding enzyme aggregation, competitive adsorption, and other limiting factors.

The catalytic activity of CH-ADS under the same experimental conditions are reported in Figure 1c. Lower values of the catalytic activity of the enzyme were observed as the initial protein concentration was increased. This can be due to different reasons:

- At higher enzyme concentrations, the immobilized enzymes can become more crowded on the surface of the support material and enzymatic multilayers can be formed [24]. This overcrowding can lead to restricted mass transfer of the substrate to the active sites of the enzymes, resulting in reduced catalytic activity. In extreme cases, the substrate molecules may have difficulty accessing the active sites altogether, leading to a significant decrease in activity.
- Higher enzyme concentrations can promote the aggregation of immobilized enzymes on the support surface [25]. Aggregation can lead to decreased accessibility of active sites and hinder substrate binding, reducing the overall catalytic activity.
- When enzyme concentration is increased, the probability of multiple enzyme molecules simultaneously binding to the same substrate molecule (substrate competition) also increases [27]. This can lead to a decrease in the effective number of enzymes available for catalysis, thus reducing the overall activity.
- High enzyme concentrations can lead to conformational changes in the immobilized enzymes [28], altering their active site structure and reducing their catalytic efficiency.
- In some cases, high enzyme concentrations can lead to enzyme deactivation or denaturation on the support surface, especially if the immobilization process involves harsh conditions [24].

To optimize the catalytic activity of immobilized enzymes, it is essential to carefully control the enzyme concentration during the immobilization process. Balancing the enzyme concentration with the support material, immobilization method, and reaction conditions is crucial for achieving the best catalytic performance. For this reason, the subsequent experiments with CH-ADS were carried out adopting an initial lipase concentration of 6 mg/mL as a reasonable compromise between these conflicting factors.

**Figure 2:**
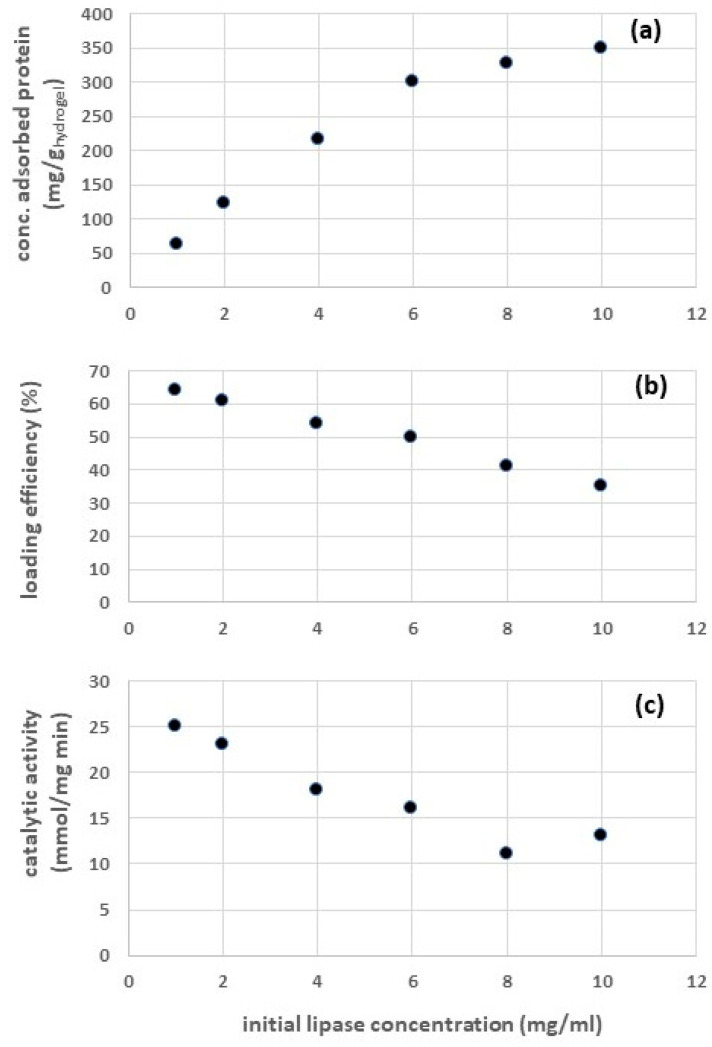
Results of lipase entrapment in chitosan hydrogel particles at different values of the initial lipase concentration: (**a**) concentration of the entrapped enzyme, (**b**) loading efficiency, and (**c**) catalytic activity.

Similar experiments were carried out with reference to the chitosan hydrogel containing entrapped lipases (CH-ENT). The results obtained are reported in Figure 2. A comparison between Figure 1a,b and Figure 2a,b shows that, when entrapping the enzyme, both the concentration of the immobilized lipase and the loading efficiency are higher. This result can be explained by observing that entrapment involves physically trapping the enzyme molecules within a matrix or gel structure, whereas adsorption involves binding the enzyme molecules onto the surface of an adsorbent support. The concentration of immobilized enzymes in CH-ENT is generally higher compared to that observed in CH-ADS for various reasons:

- In entrapment, the enzyme molecules are physically enclosed or embedded within a porous matrix or gel. This matrix provides a three-dimensional network that can hold a significant amount of enzymes, leading to a higher concentration of entrapped enzymes within the matrix [29].
- The entrapment process usually has a higher retention capacity, as the enzyme molecules are held securely within the matrix. This allows for a more efficient use of the available enzymes, resulting in a higher concentration of entrapped enzymes compared to the adsorbed enzymes on the surface of an adsorbent support [29].
- The mechanism of entrapment involves encapsulating the enzyme within the support material, while adsorption typically involves surface binding. Entrapment allows for higher enzyme-to-support interactions, leading to higher enzyme concentration [29].
- In certain cases, adsorbed enzymes may be prone to leaching out of the matrix over time, reducing the overall enzyme concentration and, consequently, the catalytic activity [30].

Nevertheless, entrapment may not always be the best immobilization method for certain reactions or processes, and other factors, such as enzyme accessibility, substrate diffusion, and enzyme loading efficiency, also play crucial roles in determining the overall catalytic efficiency and performance of immobilized enzymes.

However, by comparing Figure 1c and Figure 2c, it can be observed that the catalytic activity of CH-ENT is lower in comparison to that of CH-ADS. This result can be due to various reasons:

- When the enzyme is entrapped, it is almost uniformly distributed within the chitosan particles (whereas the adsorbed lipases are mainly concentrated on the external surfaces). Consequently, entrapped enzymes may experience limited diffusion of substrates and products within the porous matrix, leading to reduced access to the enzyme’s active sites. This mass transfer limitation can hinder the catalytic efficiency of the entrapped enzyme compared to the adsorbed enzyme, which has direct contact with the surrounding medium.
- The confinement of enzymes within the matrix can affect their conformation and flexibility, potentially leading to reduced catalytic activity [28]. Some enzymes may require a certain degree of freedom in their structure to function optimally, and confinement can restrict their movement and negatively impact their activity.
- The immobilization process can influence the enzyme’s interaction with the support material. The entrapment process may alter the enzyme’s microenvironment, affecting its catalytic activity [31].
- During the enzyme entrapment procedure, a lipase solution was added dropwise into a solution containing methanol (see Section 4.3). Obviously, the methanol-induced inactivation may affect the catalytic activity of the entrapped enzyme [32].

In order to provide evidence that the immobilization procedures adopted actually lead to enzyme adsorption (in CH-ADS samples) and entrapment (in CH-ENT samples), we first obtained SEM images of the solid supports (Figure S2). No significant differences were observed between the SEM images of the chitosan hydrogel without enzyme, the CH-ADS, and the CH-ENT samples.

**Figure 3:**
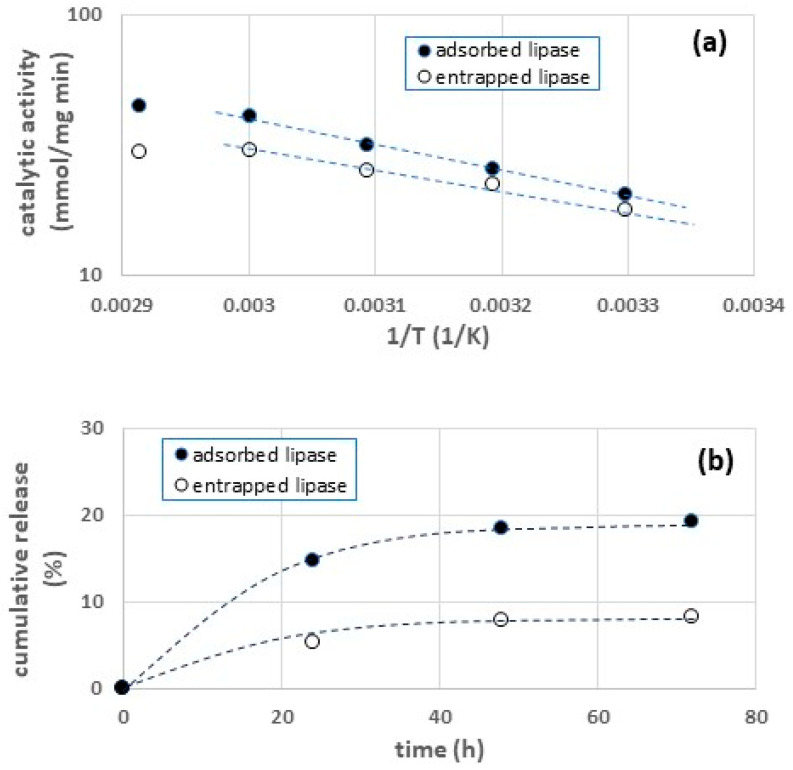
Arrhenius plot (**a**) and release kinetics (**b**) of immobilized lipase.

Consequently, we adopted two experimental approaches to discriminate between adsorption and entrapment phenomena:

First, we carried out kinetic tests at different temperatures to measure the activation energies (E_att_). The Arrhenius plots of CH-ADS and CH-ENT are shown in Figure 3a. The values of E_att_ obtained by nonlinear regression are 19.0 Kcal/mol for CH-ADS and 14.5 Kcal/mol for CH-ENT. The lower value of E_att_ for CH-ENT is due to the effect of the mass transfer resistances on the overall reaction kinetics, as the transport phenomena depend weakly on the temperature [33]. This result confirms that the enzymes immobilized in the CH-ENT samples are mainly entrapped and that, as already observed, the entrapment of enzymes may lead to limited diffusion of substrates and products within the porous matrix, thus affecting the apparent reaction kinetics.

Secondarily, we measured the kinetics of desorption of the enzyme in a water solution. The results in Figure 3b show that enzyme release from the CH-ADS samples is significantly faster. This result demonstrates that the enzymes immobilized in the CH-ADS samples are mainly adsorbed. As a matter of fact, adsorption typically involves surface binding of the enzyme, which favors the desorption, while entrapment allows the segregation of the enzyme within the support material.

It is worth noting that most industrial applications of lipases are carried out in nonaqueous media or even in solvent-free systems [34]. Under these conditions the enzyme release will be significantly slower, as the solubility of proteins in nonaqueous media is usually reduced.

### 2.2. Stability of Immobilized Lipase in the Presence of Triglyceride Oxidation Products

The subsequent experiments were aimed at studying the influence of different triglyceride oxidation products on the operational stability of the lipases in a nonaqueous environment.

**Figure 4:**
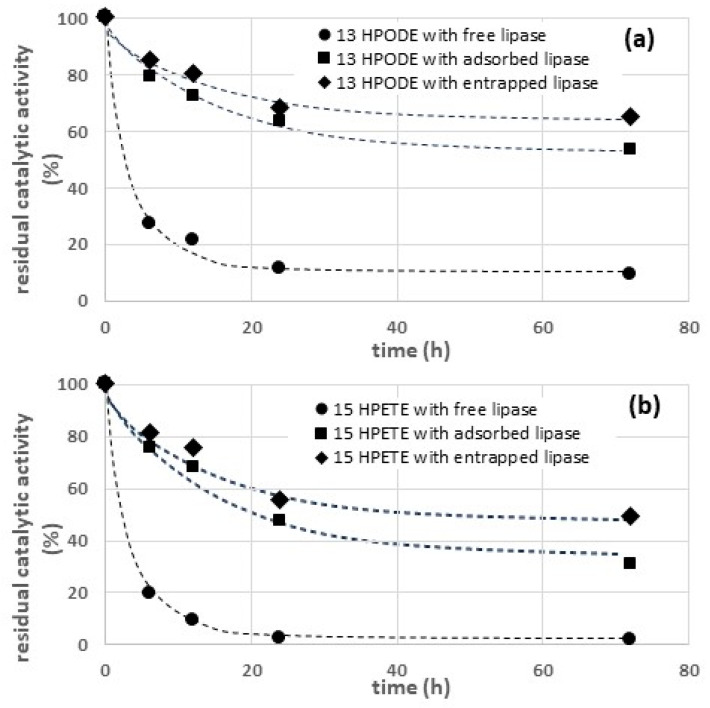
Effect of two hydroperoxides on thermal stability of free and immobilized lipase at T = 50 °C: (**a**) 13-HPODE and (**b**) 15-HPETE.

First, the stability of lipases was characterized in the presence of two hydroperoxides, i.e., intermediate products of triglyceride oxidation. In this view, 15-HPETE and 13-HPODE, obtained by controlled oxidation of arachidonic and linoleic acid, were characterized as regards their inactivating effect. The results are reported in Figure 4. All the inactivation curves shown in Figure 4 could be successfully interpolated via adopting an exponential model:

(1) $$R=A\cdot e^{-kt}+B$$

where *R* is the residual catalytic activity (%) and *t* is the time (h). The parameters obtained by nonlinear interpolation of the experimental curves are reported in Table 1.

Comparing Figure 4a,b, and considering the data in Table 1, it is possible to observe that the asymptotic level of the residual catalytic activity (represented by the parameter B) is much lower in the presence of 15-HPETE than in the presence of 13-HPODE.

The experimental curves reported in Figure 4a indicate that both the adsorbed and the entrapped lipases are significantly more stable compared to the free enzyme. A similar conclusion can be drawn from the curves in Figure 4b.

The data in Table 1 also show that the asymptotic level of the residual catalytic activity observed with the entrapped enzyme is higher in comparison to the adsorbed enzyme, both in the presence of 15-HPETE and in the presence of 13-HPODE. The higher stability of entrapped enzymes can be due to different factors:

- Entrapped enzymes, being physically confined within a solid matrix, are more protected from inactivating factors, both physical and chemical [23].
- Being confined within a matrix, entrapped enzymes have a lower tendency to detach from the solid support [30].
- Entrapped enzymes experience limited conformational changes from their native configuration, as the entrapment restricts the enzymes’ movement [28].

To characterize the effect of the final products of triglyceride oxidation, stability tests were carried out using a series of compounds structurally related to the secondary products of lipid peroxidation. In this view, we selected four model compounds with a similar length of the hydrocarbon chain, representing different classes of secondary oxidation products: an alkene (2-hexene), an aldehyde (2-hexenal), an allylic alcohol (2-hexen-1-ol), and a carboxylic acid (2-hexenoic acid).

**Figure 5:**
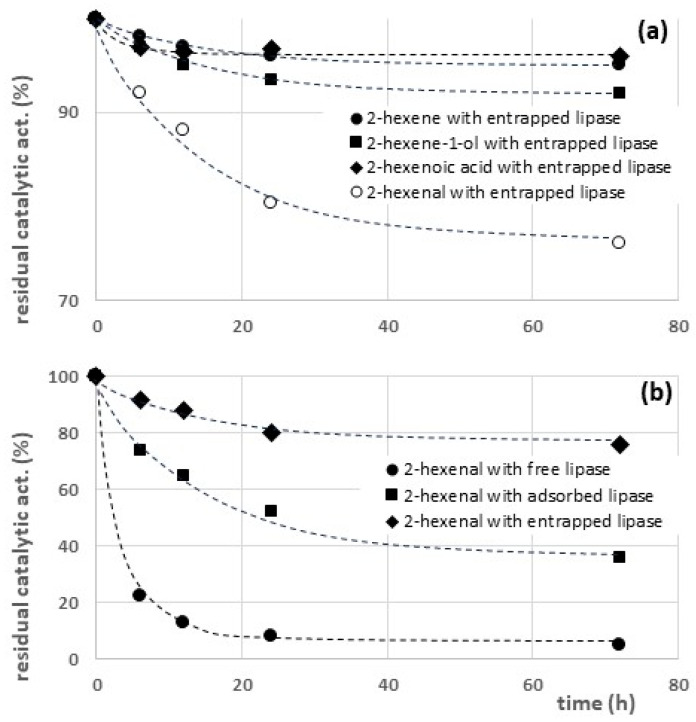
Effect of secondary products of triglyceride oxidation on thermal stability of free and immobilized lipase at T = 50 °C: (**a**) effect of sample secondary products and (**b**) effect of 2-hexanal.

These model compounds were characterized as regards their effect on the stability of the entrapped lipase. The results shown in Figure 5a demonstrate that the aldehyde 2-hexanal generates the most significant inactivating effect.

Further experiments were then carried out to characterize the inactivating effect of 2-hexanal on the free lipase, the adsorbed lipase, and the entrapped lipase. The results reported in Figure 5b confirm that, even in the presence of the secondary products of triglyceride oxidation, the entrapped lipase is more stable than the adsorbed lipase.

To summarize the results obtained, the catalytic activity of the entrapped lipase (CH-ENT) is lower in comparison to that of the adsorbed enzyme (CH-ADS), whereas the stability of CH-ENT in the presence of the products of the triglyceride oxidation is higher. It is worth noting that both these results can be explained considering the reduced mass transfer through the hydrogel matrix. As a matter of fact, the lower catalytic activity of the CH-ENT is caused by the limitations of the mass transfer of substrates and products, whereas the adsorbed enzyme (CH-ADS) has direct contact with the surrounding medium.

On the other hand, the porous matrix also reduces the contact of the entrapped lipases (CH-ENT) with the inactivating compounds (i.e., hydroperoxides and aldehydes), ensuring a long-term stability higher in comparison to that of the adsorbed enzyme (CH-ADS). The difference between the long-term stability of CH-ENT and CH-ADS is particularly significant in the presence of 2-hexanal (Figure 5b), probably due to the partially polar character of the aldehyde (log P = 1.79), causing hydrophobic repulsion with the hydrophilic functional groups of the chitosan hydrogel, and consequently, a more effective protection of the enzyme. On the contrary, due to the hydrophobic character of 13-HPODE (log P = 6.0) and 15-HPETE (log P = 5.8), the penetration of these compound is easier, and consequently, the stabilization of the entrapped enzyme in the presence of these compounds is only partial (Figure 4a,b).

## 3. Conclusions

A lipase from *Candida rugosa* has been immobilized on a chitosan hydrogel following two alternative approaches based on enzyme adsorption and entrapment, respectively. When the enzyme was entrapped, both the concentration of the immobilized lipase and the loading efficiency were higher, though the specific activity of the entrapped enzyme was lower due to a higher resistance to the mass transfer of substrates and products.

The entrapped lipases showed a lower value of activation energy in comparison to the adsorbed lipase, demonstrating that the entrapment of the enzyme causes mass transfer resistances affecting the overall reaction kinetics. However, the entrapped lipases showed a slower kinetics of desorption due to the complete segregation of the enzyme within the solid support.

The immobilization of chitosan hydrogels has significantly increased the stability of the enzyme in the presence of the products of triglyceride oxidation, both primary (the hydroperoxides 15-HPETE and 13-HPODE) and secondary (2-hexene, 2-hexenal, 2-hexen-1-ol, and 2-hexenoic acid). A nonaqueous reaction medium has been adopted for kinetic tests to reproduce the operating conditions that are typical of most oleochemical processes. When testing the long-term stability of the enzyme in the presence of hydroperoxides, the entrapped lipase was in all cases more stable. The same result was obtained when measuring the inactivating effect of secondary products of triglyceride oxidation, though aldehydes proved to be the most inactivating class of products.

## 4. Materials and Methods

### 4.1. Materials

Low molecular-weight chitosan (average molecular weight 110,000; average deacetylation degree 75%), lipase from *Candida rugosa*, geraniol, ethyl caproate, and all the remaining reactants were purchased from Sigma-Aldrich. All reactants used were analytical grade.

The 15-hydroperoxy-5,8,11,13-eicosatetraenoic acid (15-HPETE) and the 13-hydroperoxy-9,11-octadecanoic acid (13-HPODE) were obtained following specific synthetic protocols [35], by enzymatic oxidation of arachidonic acid (2 mM) or linoleic acid (3 mM) with soybean lipoxygenase type 1B (8300 U/mL) in 0.1 M pH 9.0 borate buffer (1 L). The concentrations of the acids were measured spectrophotometrically at 234 nm, adopting the following extinction coefficients: 23.3·10^3^ for 15-HPETE [36] and 27.0·10^3^ for 13-HPODE [37].

The malondialdehyde (MDA) was prepared by acid hydrolysis of 1,1,3,3-tetraethoxypropane. The 4-Hydroxynonenal (4-HNE) was purchased from Cayman chemicals as an ethanol solution.

### 4.2. Synthesis of the Chitosan Hydrogel

A solution of chitosan was prepared by dissolving chitosan (2% *w*/*v*) in a 1% (*v*/*v*) acetic acid solution. The chitosan was completely dissolved by stirring for 3 h. The solution obtained was then added dropwise, using a 1 mm diameter syringe, into a solution of methanol in HCl (20:1 *v*/*v*) containing 1% glutaraldehyde. The hydrogel beads obtained were removed by filtration and used for adsorption experiments.

In order to confirm that a chitosan hydrogel was obtained, the swelling degree was measured, keeping a sample of chitosan particles in an aqueous solution of acetic acid (pH 5) at 37 °C until constant dry weight. The swelling degree, measured as the ratio between the initial and final weights of the swollen samples, was 55 ± 2.6%, in agreement with the values reported in previous studies.

The formation of the chitosan hydrogel was confirmed by the results of the FTIR analysis of both the chitosan and the chitosan hydrogel, described in the Figure S1. A detailed comparison of the FTIR spectra demonstrated the formation of an imine group (C=N) formed by covalent bonding between the free amino groups of chitosan and the aldehyde groups of glutaraldehyde.

### 4.3. Synthesis of the Chitosan Hydrogel with Entrapped Lipase (CH-ENT)

A solution of chitosan (2% *w*/*v*) in 8 mL of 1% (*v*/*v*) acetic acid solution was first prepared, dissolving the chitosan by stirring for 3 h. Meanwhile, a lipase solution was prepared, dissolving a given amount of *Candida rugosa* lipase (EC 3.1.1.3) in 2 mL of 1% (*v*/*v*) acetic acid solution. The two solutions were then mixed. The solution obtained was added dropwise, using a 1 mm diameter syringe, into a solution of methanol in HCl (20:1 *v*/*v*) containing 1% glutaraldehyde. The hydrogel beads obtained were removed by filtration, washed three times with Na-acetate buffer to remove weakly adsorbed enzymes, and then used for adsorption experiments. It is worth noting that bifunctional glutaraldehyde molecules may interact with the amino groups of the lipase, leading to a partial cross-linking of the enzyme. Consequently, an apparent increase in the entrapment loading efficiency could be due to the interaction between glutaraldehyde and lipase.

### 4.4. Chitosan Hydrogel with Adsorbed Lipase (CH-ADS)

The adsorption protocol was started by dissolving a given amount of lipase in a Na-phosphate buffer solution (40 mL). Then, chitosan particles (400 mg) were added to the solution. The solution was kept under gentle stirring (60 rpm) for 24 h at 4 °C, until the adsorption equilibrium was reached, as ascertained by preliminary tests. The chitosan particles were then separated by vacuum filtration and washed three times with Na-acetate buffer to remove weakly adsorbed enzymes and dried at room temperature.

An amount of excess glutaraldehyde, resulting from the previous hydrogel synthesis, could be present on the hydrogel particles surface. Consequently, an apparent increase in the adsorption loading efficiency could be due to the interaction between glutaraldehyde and lipase.

### 4.5. Measurement of Lipase Concentration, Release, Catalytic Activity, and Activation Energy

The lipase concentration was measured by the method of Bradford [38], recording the absorbance at 595 nm. Bovine serum albumin was used as reference protein.

The loading efficiency (LE) was calculated as the ratio between the amount of enzyme immobilized on the support and the total amount of enzyme used:

(2) $$LE=\frac{W_{0}-W_{1}}{W_{0}}$$

where *W*_0_ is the total weight of enzyme initially added to the solution and *W*_1_ is the weight of enzyme not bounded to the support.

The release of lipases from the hydrogel particles was assessed by incubating the biocatalyst in Na-phosphate buffer (pH 7.0) under orbital mixing (300 RPM). At given times, samples of the supernatant were collected to evaluate the lipase concentration by the Bradford method.

The catalytic activity of the immobilized lipase was measured via carrying out the synthesis of geranyl caproate by transesterification of geraniol and ethyl caproate. In a typical test, a given amount of lipase-based biocatalyst was added to 2 mL of octane solution containing 100 mM geraniol and 100 mM ethyl caproate. The reaction was usually carried out in an orbital stirrer (100 rpm) at 37 °C. The samples collected after each test were diluted and analyzed by gas–liquid chromatography (GLC). A gas chromatograph (Shimadzu CC 17/3) with an FID detector was used, equipped with a flame ionization detector. Helium was used as carrier gas at a constant temperature of 160 °C. The enzyme activity was defined as activity per milligram of dry catalyst (millimoles per milligram per minute).

The activation energies of the immobilized lipases were measured by carrying out the enzymatic assay previously described at different temperatures. The activation energy was measured in the temperature range between 30 °C and 70 °C.

### 4.6. Stability of Lipases in the Presence of Triglyceride Oxidation Products

In the stability tests, the methanol solution (0.1 mL) of a given oxidation product was mixed with pure soybean oil (2 mL) and shaken at 50 °C until all the solvent had evaporated. The mixture was then added to a sample of lipase (10 mg) and incubated at 50 °C for a given time.

The residual activity of the immobilized lipase was measured via carrying out the synthesis of geranyl caproate by transesterification of geraniol and ethyl caproate, as described in the Section 4.5.

### 4.7. Statistical Analysis

All the experiments have been carried out in triplicate. All data are presented as mean ± standard deviation (*n* = 3).

## Figures and Tables

**Table 1:** Parameters obtained by linear regression of the curves shown in Figure 4 using the mathematical model (1).

	In the Presence of 13-HPODE			In the Presence of 15-HPETE		
	A	k (h^−1^)	B	A	k (h^−1^)	B
Free lipase	88.0	2.70 × 10^−1^	11.8	97.1	2.87 × 10^−1^	2.77
CH-ADS	44.7	7.92 × 10^−2^	54.1	66.3	5.61 × 10^−2^	29.4
CH-ENT	35.4	8.08 × 10^−2^	64.4	52.3	6.72 × 10^−2^	47.7

## Supplementary Materials

The following supporting information can be downloaded at: https://www.mdpi.com/article/10.3390/gels9100776/s1, Figure S1: Fourier Transformed Infrared Spectroscopy (FTIR) spectra of chitosan (a) and chitosan hydrogel (b) and Figure S2: SEM micrographs of the chitosan hydrogel, without enzyme (a), with adsorbed lipase (b) and with entrapped lipase (c). Magnification: 100× (scale 0.5 mm).
