# Supplementary material for: Immobilization of Lipases on Chitosan Hydrogels Improves Their Stability in the Presence of the Products of Triglyceride Oxidation
Source: Gels. 2023 Sep 24;9(10):776. doi: 10.3390/gels9100776 (PMC10606435; doi:10.3390/gels9100776)
Supplement: Supplementary file 1 [file gels-09-00776-s001.zip › gels-2554935-supplementary.pdf]

SUPPLEMENTARY INFORMATION

Immobilization of lipases on chitosan hydrogels improves their stability in the presence of the products of triglyceride oxidation

S1. Characterization of the chitosan hydrogel by Fourier Transformed Infrared Spectroscopy (FTIR)

The infrared spectra of the chitosan hydrogel were obtained using a FTIR spectrometer (Thermo Nicolet, USA).

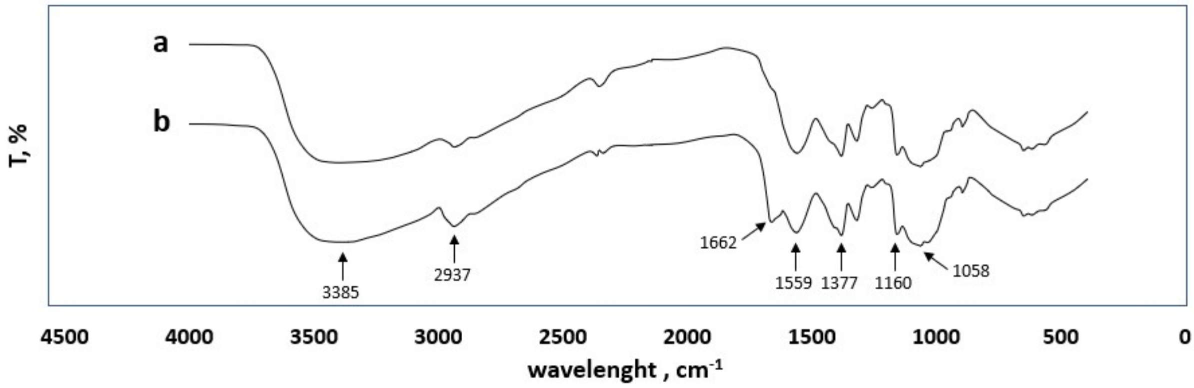

Figure S1. Fourier Transformed Infrared Spectroscopy (FTIR) spectra of chitosan (a) and chitosan hydrogel (b).

The FTIR spectra of the chitosan hydrogel, shown in the Fig. S1 (curve b), indicated the presence of the of the functional groups described in the following. The peaks (Typical bands) at 3385 cm^{-1} and 2937 cm^{-1} correspond to the stretching of O-H and of C-H, respectively. The peak at 1559 cm^{-1} can be attributed to the N–H group. The $–CH_3$ symmetrical deformation was confirmed by the peak at 1377 cm^{-1}. The peak at 1160 cm^{-1} can be attributed to the asymmetric stretching of the C-O-C bridge. The peak at 1058 cm^{-1} corresponds to the stretching of C–O group.

All these peaks can be observed to some extent also in the chitosan spectrum (curve a), that has been reported as reference. Yet, the effective formation of the chitosan hydrogel is demonstrated by the presence of the peak at 1662 cm^{-1} (not found in the curve a), corresponding to the imine groups (C=N) formed by covalent bonding between the free amino groups of chitosan and the aldehyde groups of glutaraldehyde.

S2. SEM micrographs of the chitosan hydrogel

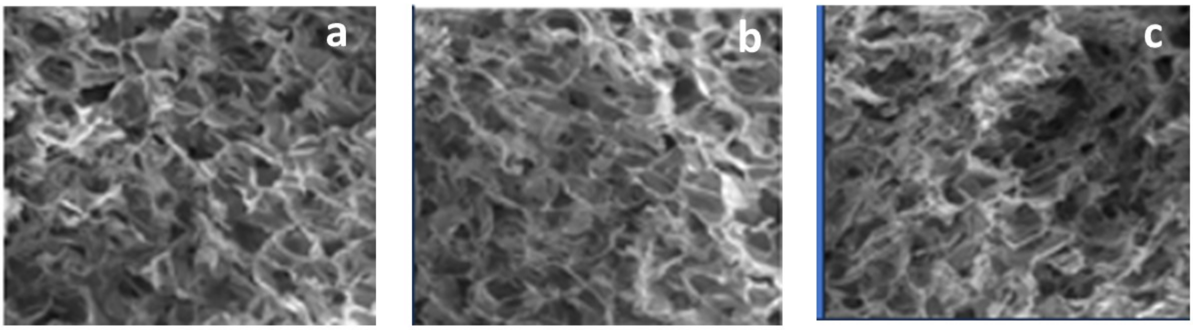

Figure S2. SEM micrographs of the chitosan hydrogel, without enzyme (a), with adsorbed lipase (b) and with entrapped lipase (c). Magnification: 100x (scale 0.5 mm).
